# Parasitoids of the eucalyptus gall wasp *Leptocybe invasa* (Hymenoptera: Eulophidae) in China

**DOI:** 10.1051/parasite/2016071

**Published:** 2016-12-21

**Authors:** Xia-Lin Zheng, Zong-You Huang, Dan Dong, Chun-Hui Guo, Jun Li, Zhen-De Yang, Xiu-Hao Yang, Wen Lu

**Affiliations:** 1 College of Agriculture, Guangxi University Nanning 530004 Guangxi Zhuang Autonomous Region PR China; 2 College of Forestry, Guangxi University Nanning 530004 Guangxi Zhuang Autonomous Region PR China; 3 Department of Guangxi Forestry Pest Management Nanning 530028 Guangxi Zhuang Autonomous Region PR China

**Keywords:** Forestry pest, Gall-inducing insect, Biological control, Parasitoids

## Abstract

*Leptocybe invasa* Fisher & La Salle (Hymenoptera, Eulophidae) is an invasive pest in *Eucalyptus* plantations throughout the world. Potential biological control agents for *L. invasa* were investigated in the Fujian, Guangdong, Hainan, Guangxi, Jiangxi, and Sichuan provinces of China, where *Eucalyptus* spp. have been severely damaged by the eucalyptus gall wasp. Three hymenopteran parasitoids of *L. invasa* were identified: *Quadrastichus mendeli* Kim & La Salle (Eulophidae), *Aprostocetus causalis* La Salle & Wu (Eulophidae), and *Megastigmus viggianii* Narendran & Sureshan (Torymidae); *M. viggianii* is newly recorded in China. The percentages of parasitization by *Q. mendeli*, *A. causalis*, and *M. viggianii* were 2.96%–19.53%, 2.30%–26.38%, and 24.93%, respectively. The longevity and body length of females were significantly greater than for males in *A. causalis* and *M. viggianii*. No males of *Q. mendeli* were found in China. These parasitoids could be used as biological agents for *L. invasa* in China.

## Abstract (fr)

*Leptocybe invasa* Fisher & La Salle (Hymenoptera, Eulophidae) est un ravageur envahissant dans les plantations d’eucalyptus dans le monde. Les agents potentiels de lutte biologique contre *L. invasa* ont été étudiés en Chine dans les provinces de Fujian, Guangdong, Hainan, Guangxi, Jiangxi et Sichuan, où les *Eucalyptus* spp. ont été gravement endommagés par la galle de l’eucalyptus. Trois hyménoptères parasitoïdes de *L. invasa* ont été identifiés : *Quadrastichus mendeli* Kim & La Salle (Eulophidae), *Aprostocetus causalis* La Salle et Wu (Eulophidae) et *Megastigmus viggianii* Narendran & Sureshan (Torymidae) ; *M. viggianii* est une nouvelle mention pour la Chine. Le pourcentage de parasitisme par *Q. mendeli*, *A. causalis* et *M. viggianii* étaient 2.96 %–19.53 %, 2.30 %–26.38 % et 24.93 %, respectivement. La longévité et la longueur du corps étaient significativement plus élevées chez les femelles que chez les mâles pour *A. causalis* et *M. viggianii*. Aucun mâle de *Q. mendeli* n’a été trouvé en Chine. Ces parasitoïdes pourraient être utilisés comme agents biologiques contre *L. invasa* en Chine.

## Introduction

Eucalyptus is one of the three major fast-growing tree species worldwide, which plays important roles in reforestation and the production of timber, pulp, potential bioenergy feedstock, and other forest products [25]. In China, the cultivated area under eucalyptus covers more than 3.68 billion hectares and produces a direct economic income that exceeds 100 billion Renminbi (RMB) [33]. However, the decline of ecosystem biodiversity is very obvious with the increased cultivated area of eucalyptus and results in a sharp rise of eucalyptus insect pests [22]. The number of eucalyptus insect pest species in China has increased from 53 in 1980 to 319 in 2011 and causes direct economic losses exceeding RMB 1.125 billion annually [23].

The eucalyptus gall wasp, *Leptocybe invasa* Fisher & La Salle (Hymenoptera: Eulophidae), originating from Australia, is a global pest in *Eucalyptus* plantations [18]. A recent study based on molecular and phylogenetic analyses suggested the occurrence of geographical variability in *L. invasa* populations and the existence of different putative species, among them a “Chinese lineage” [20]. The wasp populations investigated in our study were not characterized from a phylogenetic point of view; we therefore cannot indicate their exact taxonomical position. Moreover, as the lineage is not a taxonomic category coded by the International Code of Zoological Nomenclature, in the present paper the wasp is cited as *L. invasa*. *Leptocybe invasa* has expanded to more than 29 countries in Asia, Europe, Africa, and the Americas [18, 35]. In China, *L. invasa* was first found in the Guangxi Zhuang Autonomous Region in April 2007 [30]. Subsequently, the pest has spread to Guangdong, Fujian, Hainan, Jiangxi, and Sichuan provinces [3, 29, 31, 34]. Various management strategies have been explored to control *L. invasa*, including chemical control [13], breeding and the selection of resistant planting stock [5, 34], and biological control [15, 16]. However, chemical control is not widely accepted due to its varying success, negative effects on biodiversity, and environmental pollution. Sylvicultural control is largely ad hoc and is unlikely to represent a viable long-term solution against an increasing number and diverse range of damaging invasive pests. Biological control is considered an attractive alternative to other control methods due to its ecological and economic benefits [4].

In Australia, parasitoids play a very important role in limiting the populations of *L. invasa* [8, 15]. The introduction of natural enemies from Australia has been considered an optimal way to control the eucalyptus gall wasp in epidemic areas [15, 28]. However, only a few countries have adopted this method in view of increasing evidence of attacks against non-target hosts and the resulting threat to native biodiversity [15, 28]. Recently, several *L. invasa* parasitoids have been found in the invaded regions, e.g., India, Israel, Turkey, Italy, Sri Lanka, Thailand, Argentina, and South Africa [7–9, 12–15, 21]. However, as mentioned above, the parasitic capacities of these parasitoids for *L. invasa* are different in these regions. Thus far, only *Aprostocetus causalis* La Salle & Wu (Hymenoptera: Eulophidae) and *Quadrastichus mendeli* Kim & La Salle (Hymenoptera: Eulophidae) have been reported to parasitize *L. invasa* in the Guangxi and Hainan provinces of China [10, 17, 32]. The number of parasitoid species of *L. invasa* and their parasitic capacities in the field are unknown. Therefore, it is necessary to widely investigate biological control agents for *L. invasa* in China.

The purpose of this study was to identify possible biological control agents for *L. invasa* occurring on *Eucalyptus* spp. In this study, we investigated the species of parasitoids present in some Chinese regions.

## Materials and methods

**Figure 1.**
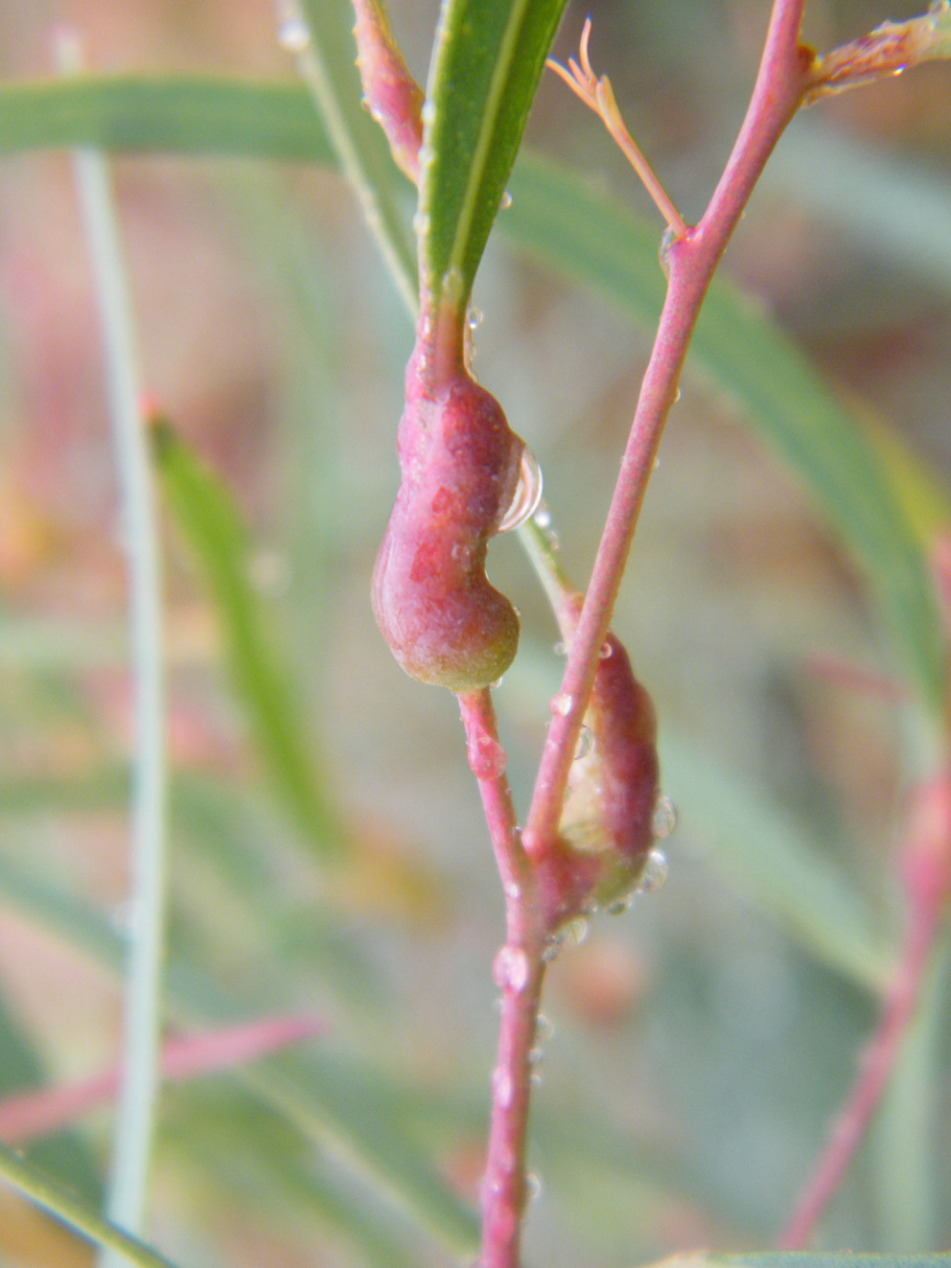
*Eucalyptus tereticornis* damaged by *Leptocybe invasa* in Guangxi.

Eucalyptus gall wasps were searched for by the typical bump-shaped galls they form on leaf midribs, petioles, and stems (Fig. 1). Branches of DH 201–2 (*Eucalyptus grandis* × *E. tereticornis*) (Myrtales: Myrtaceae), *E. tereticornis* Smith, *E. Exserta* L., and *E. grandis* × *E. urophylla* damaged by *L. invasa* were collected from Fujian, Guangdong, Hainan, Guangxi, Jiangxi, and Sichuan provinces from 2015 to 2016. The sampling sites and sampling times for each province are shown in Table 1. Branches were placed in a glass container filled with water to retain freshness and transferred to a sealed net cage (40 cm × 40 cm × 80 cm) at 27 ± 1 °C (the average air temperature of the sampling sites during the period of collection) with an L16:D8 photoperiod and 70–80% relative humidity to prevent the adults from escaping. The water in the glass container was replaced daily until the emergence of *L. invasa* and their parasitoids.

**Table 1.** Percentages of parasitization by parasitoids in *Leptocybe invasa*.

Geographical population	Sampling site	Sampling time	Family	Species	EP/EGP	Percentage of parasitization (%)
Fujian	Yongxi Village (117°95′ E, 26°48′ N), Qingzhou Town, Sha County, Sanming City, Fujian Province	25 April 2015 and 15 July 2016	Eulophidae	*Quadrastichus mendeli*	20/676	2.96
Guangdong	Gulianchong Village (111°57′ E, 22°63′ N), Luoping Town, Luoding City, Guangdong Province	28 May 2015		*Q. mendeli*	19/174	10.91
				*Aprostocetus causalis*	4/174	2.30
Hainan	Dongshan Village (109°20′ E, 19°66′ N), Baimajing Town, Danzhou City, Hainan Province	1 June and 10 July 2015		*Q. mendeli*	55/607	9.06
				*A. causalis*	19/607	3.13
Guangxi	The experimental field of Guangxi University (108° 29′ E, 22°85′ N), Nanning City, Guangxi Zhuang Autonomous Region	From April to July, 2015 and 2016£		*Q. mendeli*	1702/8714	19.53
				*A. causalis*	483/8714	5.54
Jiangxi	Jiangkou Village (114°73′ E, 25°58′ N), Fushi Town, Nankang City, Jiangxi Province	11 July 2016		*A. causalis*	1/26	3.84
Sichuan	Xiashagou Village (101°75′ E, 26°49′ N), Renhe Town, Panzhihua City, Sichuan Province	27 July 2016		*Q. mendeli*	44/762	5.77
				*A. causalis*	201/762	26.38
			Torymidae	*Megastigmus viggianii*	190/762	24.93

EP, number of emerged parasitoids; EGP, sum of the total number of emerged gall-formers and the total number of emerged parasitoids.

£ Samples were collected weekly from the experimental fields.

**Figure 2.**
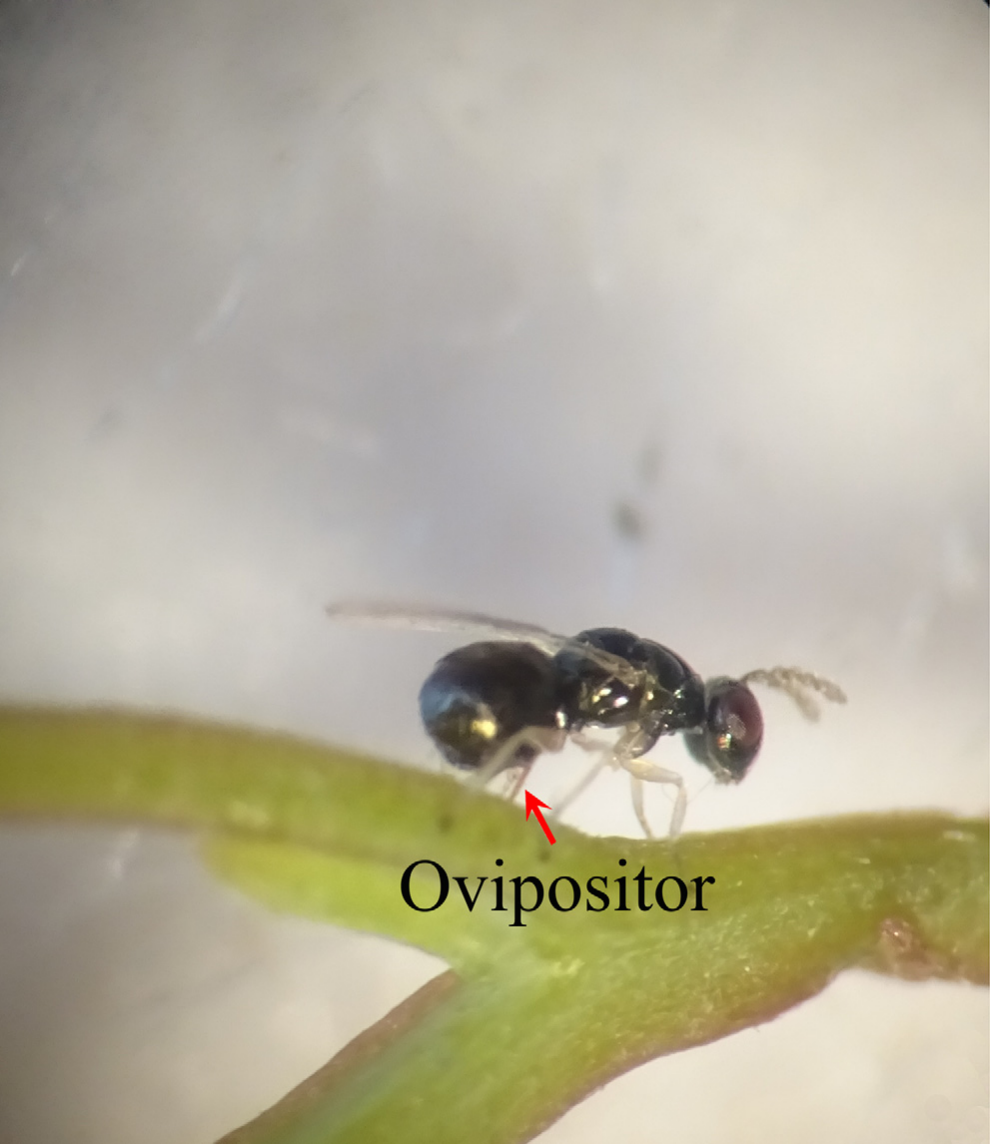
Newly emerged female of *Leptocybe invasa* inserting ovipositor into the petiole of *Eucalyptus grandis* × *E. tereticornis*.

The emerged *L. invasa* adults (Fig. 2) and their parasitoids were collected daily using 50 mL plastic tubes. The percentage of parasitization for each parasitoid collected from different geographical populations was calculated as the number of emerged parasitoids (EP) divided by the sum of the total numbers of emerged gall-formers and emerged parasitoids (EGP) [15, 16].

A cotton ball soaked in a 10% sucrose solution and galled eucalyptus branches were supplied to allow oviposition by the parasitoid adults. Newly emerged female wasps were reared one by one for uniparental species and in pairs for biparental species. The honey-water and galled branches were renewed daily. The rearing conditions of these parasitoids were similar to those of the host insects mentioned above. The mortality of the male and female specimens was recorded daily to evaluate the longevity of the parasitoids. The body lengths of these dead specimens were subsequently measured using image-measuring software (Leica Application Suite version 4.6.0, Leica Microsystems, Germany). Images of the parasitoid adults were taken with a Sony digital camera (DSC-HX60, Sony, Kyoto, Japan). Identification of the parasitoids was performed with keys [7, 15, 32] and confirmed by Prof. Chao-Dong Zhu and Dr. Huan-Xi Cao (Institute of Zoology, Chinese Academy of Sciences, China).

Statistical analysis was performed using SPSS 16.0 (SPSS, Chicago, IL, USA). Adult longevities and body lengths were compared using the nonparametric Mann-Whitney *U* test. The results were considered significant at *p* ≤ 0.05.

## Results

**Figure 3.**
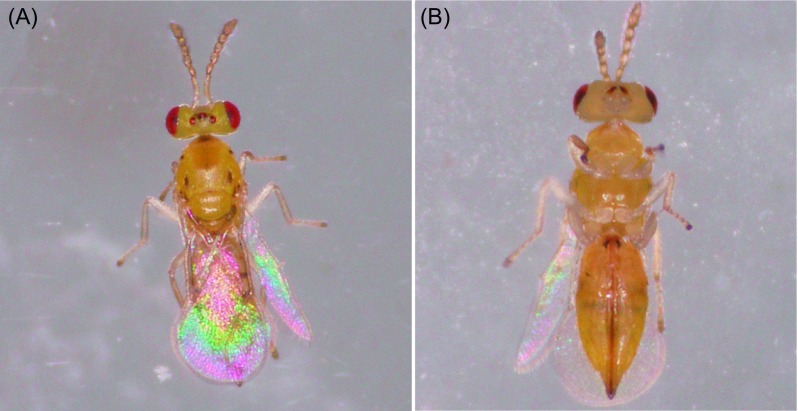
(A) Dorsal view of *Quadrastichus mendeli* female; (B) ventral view of *Q. mendeli* female.

**Figure 4.**
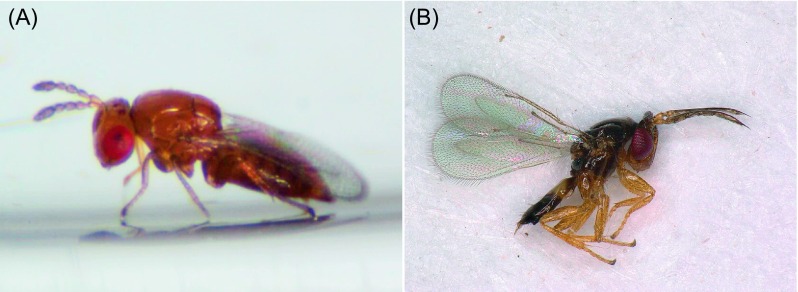
(A) Profile view of *Aprostocetus causalis* female; (B) profile view of *A. causalis* male.

**Figure 5.**
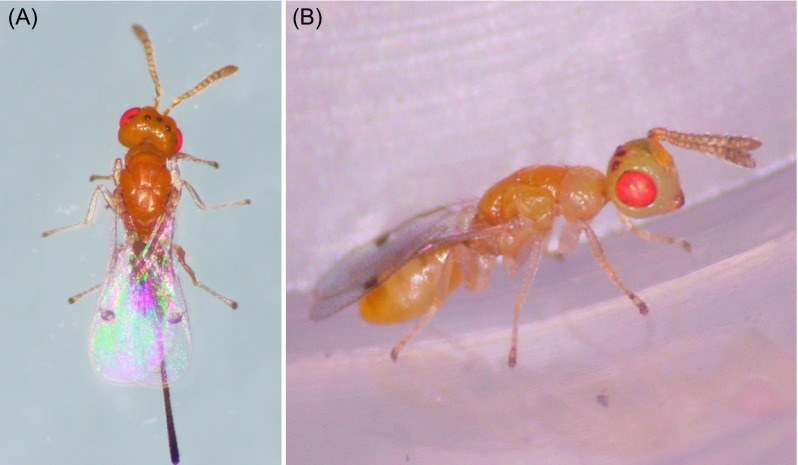
(A) Dorsal view of *Megastigmus viggianii* female; (B) profile view of *M. viggianii* male.

Three hymenopteran parasitoid species were found in *L. invasa* collected from Fujian, Guangdong, Hainan, Guangxi, Jiangxi, and Sichuan provinces: *Q. mendeli*, *A. causalis*, and *Megastigmus viggianii* Narendran & Sureshan (Hymenoptera: Torymidae); *M. viggianii* is newly recorded in China (Table 1; Figs. 3–5). The percentages of parasitization by *Q. mendeli* were 2.96, 10.91, 9.06, 19.53, and 5.77% in Fujian, Guangdong, Hainan, Guangxi, and Sichuan provinces, respectively (Table 1). No males of this species were found, and *Q. mendeli* was confirmed as a uniparental species (Fig. 3). The mean longevity and body length of the females were 5.6 ± 1.2 days and 1.2 ± 0.1 mm, respectively (Table 2). The percentages of parasitization by *A. causalis* were 2.30, 3.13, 5.54, 3.84, and 26.38% in Guangdong, Hainan, Guangxi, Jiangxi, and Sichuan provinces, respectively (Table 1). Both sexes of *A. causalis* were found (Fig. 4). The longevity (*U* = 17.0, *p* = 0.000) and body length (*U* = 20.5, *p* = 0.000) of *A. causalis* females were significantly greater than for males (Table 2). The percentage of parasitization by *M. viggianii* was 24.93% in Sichuan province (Table 1). Both sexes of *M. viggianii* were found (Fig. 5). The life span and body length of *M. viggianii* females were significantly longer than those of males (*U* = 94.0, *p* = 0.000 and *U* = 70.0, *p* = 0.000, respectively) (Table 2).

**Table 2.** Longevities and body lengths of *Quadrastichus mendeli*, *Aprostocetus causalis*, and *Megastigmus viggianii* adults.

	Sex	Longevity of adult parasitoids (days)	Body length of adults (mm)*
Eulophidae			
*Quadrastichus mendeli*	♀	5.6 ± 1.2 (30)	1.2 ± 0.1 (30)
*Aprostocetus causalis*	♂	11.5 ± 1.6 (20)	1.0 ± 0.1 (19)
	♀	15.9 ± 1.9 (20)	1.2 ± 0.1 (19)
Torymidae	♂	3.6 ± 1.0 (30)	1.4 ± 0.2 (30)
* Megastigmus viggianii*	♀	5.5 ± 1.1 (30)	2.5 ± 0.3 (30)

Values are presented as the means ±SD, with sample numbers given in parentheses.

* Body lengths of *M. viggianii* females included the ovipositor.

## Discussion

*Quadrastichus mendeli* is one of the indigenous parasitoids parasitizing *L. invasa* in Australia [15], with the percentage of parasitization varying from 7.9% to 95.6%. Recently, *Q. mendeli* was introduced from Australia to Israel and India as a biological control agent to limit the severity of damage caused by *L. invasa*. The parasitoid is now successfully established in Israel and India, and the percentage of parasitization was 73% in Israel and 81.74–94.03% in India [15, 28]. However, initial efforts to establish the *Q. mendeli* population in quarantine facilities in South Africa and Kenya have failed [6]. Interestingly, *Q. mendeli* was collected from *L. invasa* galls on *E. Camaldulensis* Dehnh. in Italy beginning in 2013, although this parasitoid was never officially released [21]. Field data showed that mean parasitization percentage by *Q. mendeli* varied from 30.2% to 50.5% in Italy [21]. Here, we report *Q. mendeli* parasitizing *L. invasa* in China under natural conditions, although this parasitoid was never officially released in China. Although *Q. mendeli* is a widely distributed *L. invasa* parasitoid in China, the percentage of parasitization differed in these geographical populations (Table 1).

*Aprostocetus causalis*, a parasitoid of *L. invasa* in China, was described as a new species in 2014 [32]. However, the longevity of both sexes of this parasitoid in the laboratory was lower than the longevity found in a previous study carried out in Thailand [27], probably due to the different rearing method. Adults in the laboratory in Thailand were fed with a honey solution, whereas both a honey solution and galled foliage were provided in this study. Previous studies suggested that parasitization involves a high physiological cost for the parasitoid and that longevity is lower in ovipositing females [24, 26].

*Megastigmus viggianii* was recorded as parasitizing the bud galls of *Calycopteris floribunda* Lamark (Myrtales: Combretaceae) in India [19]. In 2008, this parasitoid was found parasitizing *L. invasa* larvae for the first time in India [11], and the percentage of parasitization varied from 14.29% to 31.82% [28]. However, *M. viggianii* was only found in the Sichuan site; thus, we cannot ascertain whether this parasitoid is native to China. Furthermore, *M. viggianii* has a multi-host-range (i.e., *C. floribunda* and *L. invasa*) compared to *Q. mendeli* and *A. causalis*. Other *M. viggianii* hosts have not been found in China and need further investigation.

In the field, two or three parasitoids attacked *L. invasa* in China with possible competition for the monopolization of host resources. The percentage of parasitization by *Q. mendeli* was always higher than that by *A. causalis* when the two parasitoids attacked the eucalyptus gall wasp in Guangdong, Hainan, and Guangxi provinces. However, the percentage of parasitization by *Q. mendeli* was lower than those for *A. causalis* and *M. viggianii* when the three parasitoids attacked eucalyptus gall wasps in Sichuan province. We presumed that this could be attributed to the population dynamics of *Q. mendeli* in Sichuan province, but this needs to be confirmed. Previous studies showed that *Q. mendeli* is a solitary idiobiont ectoparasitoid [15] while *A. causalis* and *M. viggianii* are solitary koinobiont endoparasitoids [32]. The competitive dynamics and mechanisms of both the ectoparasitoid (*Q. mendeli*) and endoparasitoids (*A. causalis* and/or *M. viggianii*) in *L. invasa* need to be studied.

In general, parasitoids have host-tracked their hosts either simultaneously or following invasion by the pest. For example, *Ophelimus maskelli* Ashmead (Hymenoptera: Eulophidae) is a leaf-gall-inducing invasive pest of several *Eucalyptus* species in California, while its parasitoid, *Closterocerus chamaeleon* Girault (Hymenoptera: Eulophidae), was also found less than 1 year after *Q. maskelli* [1, 2]. As *Q. mendeli* and *M. viggianii* were never released in China, their record leads us to suppose that they could shortly reach other neighboring countries around China.

## Conclusion

In this study, we found that *Q. mendeli*, *A. causalis*, and *M. viggianii* could be used as biological control agents for *L. invasa* in China. However, further studies are needed on the biological characteristics, mass production and release, competitive dynamics and mechanisms of these parasitoids.

## Conflict of interest

The authors declare no conflict of interest in relation with this paper.
